# A Novel Tool for Studying Auxin-Metabolism: The Inhibition of Grapevine Indole-3-Acetic Acid-Amido Synthetases by a Reaction Intermediate Analogue

**DOI:** 10.1371/journal.pone.0037632

**Published:** 2012-05-23

**Authors:** Christine Böttcher, Eric G. Dennis, Grant W. Booker, Steven W. Polyak, Paul K. Boss, Christopher Davies

**Affiliations:** 1 CSIRO Plant Industry, Glen Osmond, South Australia, Australia; 2 The School of Molecular and Biomedical Science, University of Adelaide, Adelaide, South Australia, Australia; Griffith University, Australia

## Abstract

An important process for the regulation of auxin levels in plants is the inactivation of indole-3-acetic acid (IAA) by conjugation to amino acids. The conjugation reaction is catalysed by IAA-amido synthetases belonging to the family of GH3 proteins. Genetic approaches to study the biological significance of these enzymes have been hampered by large gene numbers and a high degree of functional redundancy. To overcome these difficulties a chemical approach based on the reaction mechanism of GH3 proteins was employed to design a small molecule inhibitor of IAA-amido synthetase activity. Adenosine-5′-[2-(1H-indol-3-yl)ethyl]phosphate (AIEP) mimics the adenylated intermediate of the IAA-conjugation reaction and was therefore proposed to compete with the binding of MgATP and IAA in the initial stages of catalysis. Two grapevine IAA-amido synthetases with different catalytic properties were chosen to test the inhibitory effects of AIEP *in vitro*. GH3-1 has previously been implicated in the grape berry ripening process and is restricted to two amino acid substrates, whereas GH3-6 conjugated IAA to 13 amino acids. AIEP is the most potent inhibitor of GH3 enzymes so far described and was shown to be competitive against MgATP and IAA binding to both enzymes with *K_i_*-values 17-68-fold lower than the respective *K_m_*-values. AIEP also exhibited *in vivo* activity in an *ex planta* test system using young grape berries. Exposure to 5–20 µM of the inhibitor led to decreased levels of the common conjugate IAA-Asp and reduced the accumulation of the corresponding Asp-conjugate upon treatment with a synthetic auxin. AIEP therefore represents a novel chemical probe with which to study IAA-amido synthetase function.

## Introduction

The auxin class of plant hormones, which is mainly represented by its ubiquitous and most abundant member indole-3-acetic acid (IAA), plays an essential role in many critical aspects of plant growth and development including embryogenesis, vascular tissue differentiation, photo- and gravitropisms, lateral branching of shoots and roots and fruit development [1]–[6]. Auxin-regulated processes depend on the tight control of the cellular auxin concentration, which requires a coordinated interplay of biosynthesis, sequestration, degradation and transport [7]–[9]. The availability of compounds that disturb this highly complex system has proven invaluable for the field of auxin transport research. The identification and functional characterisation of the membrane proteins involved in polar auxin transport has been greatly facilitated by the use of auxin transport inhibitors such as naphthylphthalamic acid, 2,3,5-triiodobenzoic acid and gravacin [10]. Chemical inhibition has also been used to assist in the study of auxin biosynthesis [11]. Kynurenine was shown to competitively inhibit a class of tryptophan aminotransferases, which have recently been established as part of a novel auxin biosynthesis pathway in Arabidopsis (*Arabidopsis thaliana* L.) [12]–[14]. Unfortunately, a compound that similarly acts to specifically inhibit protein components of metabolic pathways of auxins has not been identified to date.

The metabolic fate of auxins is poorly understood with the notable exception of the conjugation of IAA to amino acids, which is catalysed by a group of IAA-amido synthetases belonging to the family of GH3 proteins [15]–[17]. Depending on the amino acid substrate, the reaction products of these GH3 enzymes either temporarily (e.g. IAA-Ala, IAA-Leu) or permanently (IAA-Asp, IAA-Glu) remove IAA from the bioactive auxin pool [7], [8], [18], [19]. The prevalence of *GH3* genes in genomes of mosses, gymnosperms and angiosperms, [20], [21] as well as the occurrence of IAA-amino acid conjugates, in particular IAA-Asp, in most plants analysed so far [19] underlines the importance of IAA-amido synthetases for the regulation of free auxin levels. The biological function of GH3 proteins in higher plants has proven difficult to study due to large gene numbers and functional redundancy. For example, the IAA-amido synthetase group in Arabidopsis, rice (*Oryza sativa* L.) and grapevine (*Vitis vinifera* L.) consists of eight (Arabidopsis), nine (rice) and six (grapevine) members respectively and overlapping *in vitro* functions have been reported for two or more of these proteins in each of the three plant species [17], [22]–[24]. Consequently, knockout mutants of single IAA-amido synthetase genes in Arabidopsis [17], [25], [26] and rice [22] were undistinguishable from the wildtype or displayed very subtle phenotypes. However, the use of overexpression mutants has demonstrated a complex involvement of IAA-conjugating GH3 proteins in plant growth and development. In Arabidopsis, GH3–5 seems to be involved in light signal transduction pathways and stress responses [27], [28], GH3-2 and GH3-6 might have a function in the control of hypocotyl and root growth [25], [29] and for GH3-9 a role in auxin redistribution in roots has been suggested [26]. Rice overexpression mutants provided evidence for a link of GH3-8, GH3-1 and GH3-2 activities with pathogen resistance [22], [30], [31] and indicated a function of GH3-13 in drought adaptation [24]. Based on correlative evidence the activity of GH3 proteins has also been associated with fruit ripening in the pungent pepper fruit (*Capsicum chinense* Jacq.) [32] and in grape berries [23], [33]. A chemical approach targeted to inhibit the activity of IAA-amido synthetases could be used to overcome redundancy issues and the dependence on overexpression mutants provided that the inhibiting compound interacts with conserved regions in this protein family.

IAA-amido synthetases catalyse the attachment of IAA onto target substrates through two partial reactions [34]. Initially IAA is adenylated in a reaction requiring MgATP, followed by the transfer of the IAA moiety onto the amino acid substrate and the liberation of AMP. Thus, IAA-amido synthetases and GH3 proteins in general belong within a family of enzymes present in all kingdoms of life that is characterised by the employment of an adenylated reaction intermediate to attach organic acids onto substrates [35], [36]. Examples include the tRNA amino-acyl synthetases, fatty acyl ligases, biotin protein ligases and lipoyl ligase, amongst others. X-ray crystal structures for a number of these enzymes have shown that the ATP binding site is positioned juxtaposed with the binding site for the organic acid [37]–[40]. This shared structural arrangement facilitates the formation of a mixed anhydride linkage between the carboxyl group of the organic acid and the alpha phosphate of ATP. Hence, one approach towards designing a small molecule inhibitor is to create a mimic of the adenylated intermediate where the labile linker is replaced with a more stable isostere. Non-hydrolysable bi-substrate analogues have been reported as inhibitors of tRNA amino-acyl synthetase, biotin protein ligase and cysteine ligase [38], [40]–[44].

This paper describes the design and synthesis of a stable bi-substrate analogue of the GH3 reaction intermediate, adenosine-5′-[2-(1H-indol-3-yl)ethyl]phosphate (AIEP), and the characterisation of its inhibitory effects on two grapevine IAA-amido synthetases, namely GH3-1 and GH3-6. The mode of inhibition and inhibition parameters were determined using *in vitro* assays and were supported by *in vivo* data. This work provides the basis for the study of IAA-amido synthetase function through chemical inhibition.

## Results

### Recombinant Expression and Kinetic Analysis of the Grapevine GH3-6 Protein

**Figure 1:**
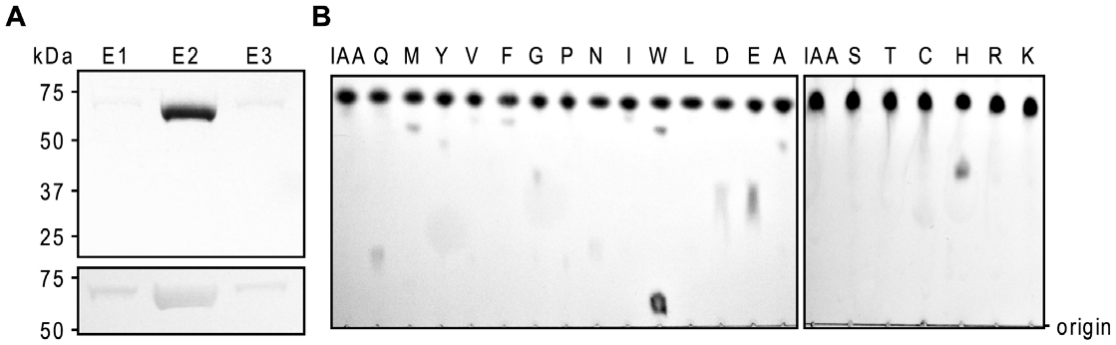
*In vitro* activity of recombinant GH3-6. (A) The expression of recombinant GH3-6 protein, which was used for the inhibition assays shown in Figs. 2 and 4, was tested by separating 10 µl of His GraviTrap column elution fractions (E1–E3) on a 4–12% polyacrylamide gel followed by Coomassie Brilliant Blue staining (upper panel) or immunodetection using a monoclonal antibody raised against poly-histidine (lower panel). The molecular mass standards (Precision Plus Protein all blue, BioRad) are indicated. The band with the size of approximately 70 kDa corresponds to the His-tagged GH3-6 protein. (B) TLC analysis of GH3-6 enzyme reactions with IAA and 20 amino acids (single letter code). The spot near the origin for the reactions with Trp represents the unbound amino acid. Plates were stained with Ehmann's reagent to detect indole compounds.

**Figure 2:**
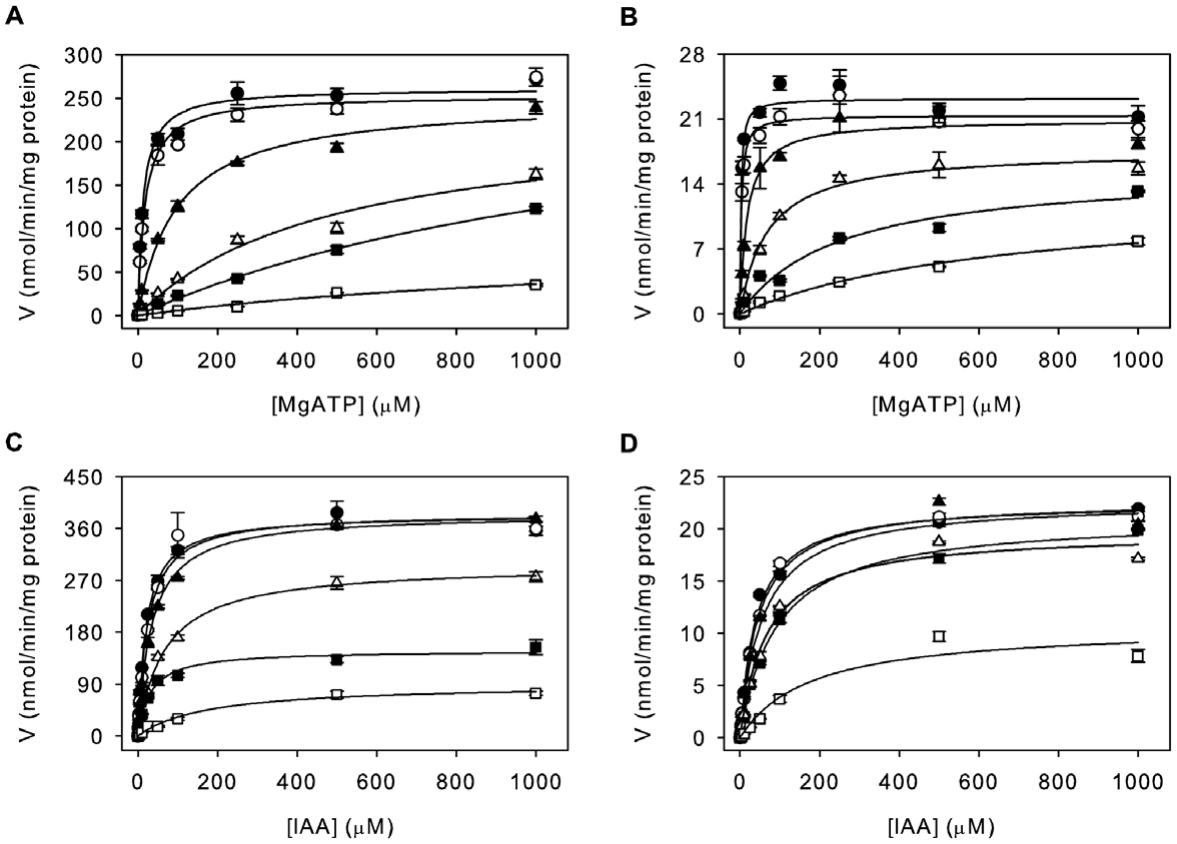
Kinetic analysis of the effect of AIEP on the binding of MgATP and IAA by GH3-1 and GH3-6. The activity of GH3-1 (A) and GH3-6 (B) was determined over a concentration range of MgATP (1 mM IAA, 1 mM Asp) by quantifying the formation of IAA-Asp after 10 min reaction time using LC-ESI-MS/MS. The same analysis was performed over a concentration range of IAA (3 mM MgATP, 1 mM Asp) using either GH3-1 (C) or GH3-6 (D). The inhibitor concentration in the reaction mix was 0 µM (•), 0.1 µM (○), 1 µM (▴), 5 µM (Δ), 10 µM (▪), or 50 µM (□). The plotted initial velocities were fitted to the Michaelis-Menten equation using non-linear regression (SigmaPlot 11.0). All data represent mean ± standard error of the mean (n = 3).

**Figure 3:**
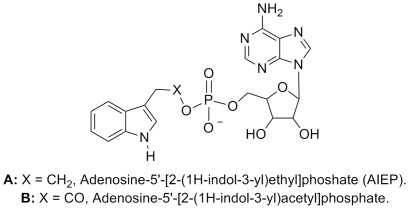
Design of AIEP. (A) gives the structure of AIEP and (B) shows the structure of the proposed reaction intermediate.

The gene encoding the putative grapevine IAA-amido synthetase GH3-6 [23] was isolated from a Cabernet Sauvignon berry cDNA template and cloned for recombinant expression in *Escherichia coli*. Introduction of a hexahistidine sequence at the C-terminus of the protein facilitated purification of the enzyme using immobilised metal ion chromatography. SDS-PAGE analysis of the protein eluted from the column revealed the enzyme preparation to be >90% pure with a single chromatography step (Fig. 1A). Initial activity tests with the purified GH3-6 protein confirmed its ability to conjugate IAA to amino acids (Fig. 1B). A thin layer chromatogram of the reaction mixtures with IAA and 20 amino acids demonstrated the possible conjugation of Gln, Met, Tyr, Val, Phe, Gly, Asn, Ile, Trp, Asp, Glu, Ala and His as indicated by a second band with lower mobility than IAA (Fig. 1B). A number of the reaction products (Glu, Met, Tyr and Ala) were tested against their corresponding IAA-amino acid standards and were found to have matching R_f_-values (data not shown). Glu, His and Trp were the preferred amino acid substrates as judged by the intensity of the product bands. The broad specificity of GH3-6 was in stark contrast to the previously characterised grapevine IAA-amido synthetase GH3-1, which has a strict requirement for just two amino acid substrates, namely Asp and Trp [33]. Next the kinetic parameters for GH3-6 were measured and compared to those of GH3-1 (Table 1, Fig. 2). The activities of GH3-1 and GH3-6 were analysed in a reaction mixture containing either 1 mM IAA, 1 mM Asp and varying concentrations of MgATP (0–1000 µM) (Fig. 2A,B) or 3 mM MgATP, 1 mM Asp and varying concentrations of IAA (0–1000 µM) (Fig. 2C,D). Asp was chosen as the amino acid substrate in all reactions since it was the preferred substrate of GH3-1 [23] and a good substrate for GH3-6 (Fig. 1B). The *K_m_*-values of 13.2 µM for MgATP and 21.1 µM for IAA were similar to those previously observed for GH3-1 [23] and the *K_m_*-values for GH3-6 were found to be 2.3 µM for MgATP and 44.7 µM for IAA (Table 1). The turnover rates for GH3-6 were approximately 10-fold lower than those measured for GH3-1, indicating that GH3-6 had a reduced catalytic efficiency when compared to GH3-1.

**Table 1:** Inhibition of GH3-1 and GH3-6 by AIEP.

Enzyme	Substrate	*K_m_* (µM)	*V_max_* (nmol min^−1^ mg^−1^)	*k_cat_* (min^−1^)	*K_i_* (µM)
GH3-1	MgATP	13.2±1.4	261.1±4.8	18	0.2
	IAA	21.1±1.4	384.3±6.2	26.5	1.2
GH3-6	MgATP	2.3±0.4	23.2±0.5	1.6	0.1
	IAA	44.7±2.5	22.8±0.4	1.6	2.7

All kinetic parameters are expressed as means ± standard error of the mean (n = 3).

### AIEP is a Competitive Inhibitor of two Grapevine IAA-amido Synthetases *in Vitro*

To address the role of IAA-amido synthetases in auxin-regulated processes an inhibitor with broad activity across all members of this enzyme family is required. Therefore, AIEP was designed to be a chemical analogue of the reaction intermediate (Fig. 3) shared by all IAA-amido synthetases. Synthesis of AIEP was achieved by coupling of 3-(2-bromoethyl)indole with AMP in the presence of sodium hydrogen carbonate (NaHCO_3_). The coupling produced several unidentified by-products, so rigorous purification of AIEP by preparative HPLC was required to obtain pure material in 15–20% yields. The chemical structure of AIEP was confirmed by NMR analysis (experimental procedures).

**Figure 4:**
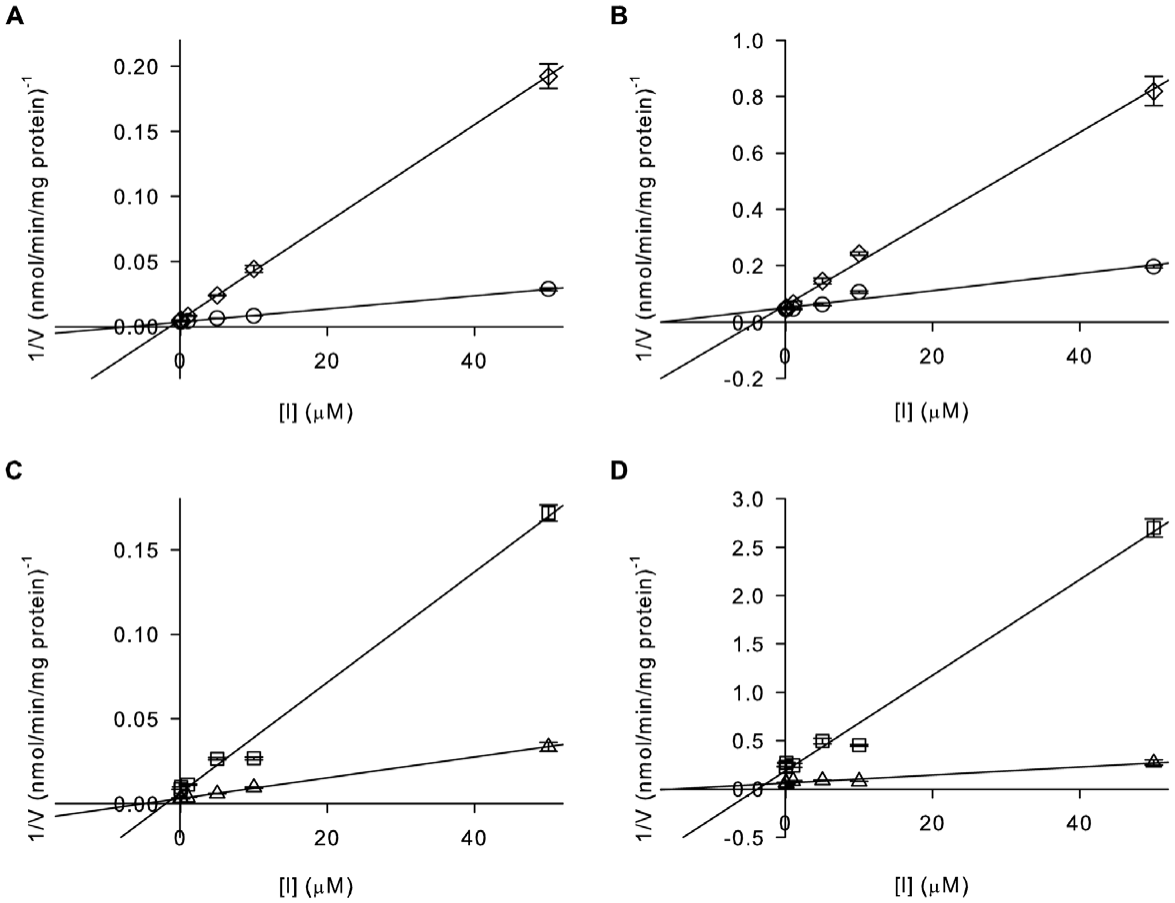
AIEP inhibits GH3-1 and GH3-6 in a competitive manner regarding both substrates – MgATP and IAA. Reciprocal initial velocities (Fig. 2) for two substrate conentrations were replotted against inhibitor concentrations. (A) Dixon plot for GH3-1 and (B) GH3-6 with varying concentrations of inhibitor and 100 µM (◊) and 1000 µM (○) MgATP and for (C) GH3-1 and (D) GH3-6 with varying concentrations of inhibitor and 10 µM (□) and 100 µM (Δ) IAA. All data represent mean ± standard error of the mean (n = 3).

The bi-substrate inhibitor was then assayed for inhibitory activity against both GH3-1 and GH3-6 to test its spectrum of activity. Reaction conditions were as described above, but included varying concentrations of the inhibitor (0.1–50 µM) in combination with varying concentrations of MgATP (Fig. 2A,B) or IAA (Fig. 2C,D). The Michaelis-Menten plots shown in Fig. 2 illustrate that AIEP reduced the activity of both grapevine GH3 proteins in a concentration-dependent manner. Consistent with a competitive inhibitor that occupies the same binding sites as ATP and IAA *V_max_*-values were unchanged with increasing inhibitor concentrations but the *K_m_*-values progressively increased. Dixon plots were performed to confirm the mechanism of inhibitor action using a selection of this data (Fig. 4). This analysis revealed that the inhibitor was indeed competitive with both IAA and MgATP with *K_i_*-values of 0.2 µM for GH3-1/MgATP, 1.2 µM for GH3-1/IAA, 0.1 µM for GH3-6/MgATP and 2.7 µM for GH3-6/IAA (Table 1). Together these data were in agreement with the initial proposal of AIEP being a mimic of the IAA-amido synthetase reaction intermediate.

### Treatment with AIEP Reduced Auxin-Asp Conjugate Formation in Grape Berries

**Figure 5:**
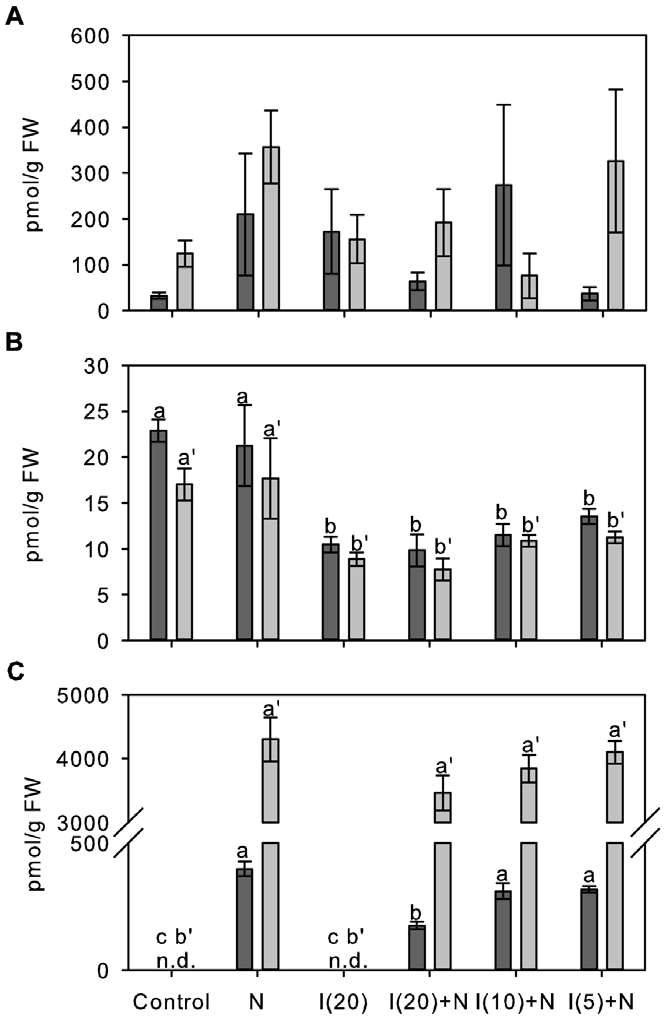
Effect of AIEP on auxin levels and the formation of auxin-Asp conjugates in Shiraz berries. (A) IAA, (B) IAA-Asp and (C) NAA-Asp were quantified by LC-ESI-MS/MS in *ex planta* Shiraz berry tissues 5 weeks prior to the initiation of ripening which had been exposed to a Control solution, 0.5 µM NAA (N), 20 µM inhibitor (I(20)) and 0.5 µM NAA in combination with 20 µM (I(20)+N), 10 µM (I(10)+N) or 5 µM (I(5)+N) inhibitor. For each treatment 20 berries were placed on 0.8% agar plates containing the indicated compounds and the plates were kept in the dark at room temperature for 6 h (dark grey bars) or 24 h (light grey bars). FW, fresh weight; n.d., not detected. All data represent mean ± standard error of the mean (n = 3). In each subfigure, bars denoted by a different letter differ significantly (p<0.05) using one-way ANOVA to compare the means followed by Duncan's post hoc test (a–c, 6 h; a′–b′, 24 h).

In order to analyse the *in vivo* effects of AIEP, Shiraz berries at about five weeks prior to the initiation of ripening were exposed to 0.5 µM 1-naphthalene acetic acid (NAA), 20 µM inhibitor or combinations of 0.5 µM NAA and three different inhibitor concentrations (5 µM, 10 µM and 20 µM) for either 6 h or 24 h in an *ex planta* experiment. Changes in the levels of the free endogenous auxin IAA, the *in vitro* GH3 reaction product IAA-Asp as well as the conjugate of Asp with the synthetic auxin NAA were analysed using LC-ESI-MS/MS. A synthetic auxin was chosen to judge the inhibitory effect of AIEP on the conjugation of an exogenous auxin of a known concentration. NAA has previously been reported as an *in vitro* acyl substrate of two grapevine IAA-amido synthetases including GH3-1 [23] and was also found to be conjugated by GH3 proteins from Arabidopsis [17] and rice [34]. None of the treatments had a significant effect on the concentration of IAA in the berry tissue (Fig. 5A), which was found to be in a comparable range to previous studies [33], [45]. The concentration of IAA-Asp (Fig. 5B) in the Control berries was also in good agreement with previously published data [33] and was not affected by the exposure to NAA for 6 h or 24 h. Treatment with 20 µM inhibitor resulted in a 2-fold decrease in the IAA-Asp concentration at both time points independent of an additional treatment with NAA and indicative of an inhibitory effect of AIEP on *in vivo* GH3 activities. Lower inhibitor concentrations (10 µM and 5 µM) led to a comparable reduction in IAA-Asp levels (Fig. 5B). A NAA-Asp concentration of 400±30 pmol/g fresh weight (FW) was measured 6 h after berries were exposed to NAA. NAA-Asp levels were about 11-fold higher (4300±340 pmol/g FW) after 24 h (Fig. 5C). The additional treatment with 20 µM inhibitor resulted in a 2.2-fold reduction in the accumulation of NAA-Asp after 6 h which matched the observed decrease in IAA-Asp levels (Fig. 5B,C). At the later time point (24 h) no significant effect of AIEP on the concentration of NAA-Asp in the berry tissue was detected. Exposure to the lower inhibitor concentration of 10 µM and 5 µM did not lead to significant changes in NAA-Asp conjugate levels at either of the two time points (Fig. 5C).

## Discussion

The conjugation of IAA to amino acids, catalysed by a group of GH3 proteins [17], has been recognised as an important aspect in the control of auxin levels in plants [7], [8], [18], which is implicated in a diverse range of processes including fruit ripening [32], [33]. A high degree of functional redundancy and large gene numbers have made the elucidation of the biological function of GH3 proteins a difficult task. The absence or subtlety of phenotypes in loss of function mutants of Arabidopsis [17], [25], [26], [28] and rice [22] illustrates this point and has led to a strong reliance on *GH3*-overexpressing mutants for functional studies which carries the risk of misinterpreting gene function due to pleiotropic effects. A specific, dose-dependent, chemical inhibition of GH3 activities would circumvent this problem and allow a broader analysis of the IAA-amido synthetase family without the time consuming generation of multiple and potentially lethal mutants.

To this end, we designed and synthesised a chemical inhibitor proposed to have broad activity towards auxin-conjugating members of the GH3 enzyme family. This compound, a non-hydrolysable analogue of the adenylated reaction intermediate, was shown to be a bi-substrate inhibitor with activity against both GH3-1 and GH3-6 *in vitro*.

A detailed kinetic analysis of a rice GH3 protein (GH3-8) has recently established a Bi Uni Uni Bi Ping Pong mechanism of catalysis with the binding of MgATP followed by IAA, the formation of an adenylated intermediate and the subsequent binding of the amino acid (Asp) [34]. Based on the high sequence similarity of plant IAA-amido synthetases [17], [20], [23] the same reaction mechanism was assumed for the two GH3 proteins used in this study, which therefore focused on the inhibition of the binding of MgATP and IAA in the first half of the reaction. The kinetic parameters obtained for the formation of IAA-Asp by GH3-1 and GH3-6 revealed that both enzymes had *K_m_*-values in the low micromolar range for both substrates (Fig. 2, Table 1) with about 2-fold (GH3-1) or 20-fold (GH3-6) higher affinities for MgATP than IAA. Substrate-velocity experiments indicated that AIEP is a competitive inhibitor of MgATP and IAA binding to the catalytic site of both GH3 proteins (Figs. 2, 4). As expected from an inhibitor which interferes with the first half of the GH3 reaction the potential differences regarding the amino acid binding site of GH3-1 and GH3-6 did not affect the mode or potency of inhibition. *K_i_*-values in the low micromolar range (Table 1) showed that AIEP inhibited the activity of GH3 proteins at least 100-fold more effectively than methyl- and ethyl-IAA, the only other compounds with a documented inhibitory effect on GH3 proteins [34]. The *K_i_*-values were 23-fold (GH3-6) and 68-fold (GH3-1) lower than the *K_m_*-values for MgATP and 17-fold (GH3-6 and GH3-1) lower than the *K_m_*-values for IAA suggesting that AIEP bound the catalytic site of the enzymes more tightly than the substrates. The 6-fold (GH3-1) and 27-fold (GH3-6) higher *K_i_*-values for IAA when compared to MgATP (Table 1) were indicative of a reduced affinity of the enzymes for the inhibitor once MgATP was bound to the catalytic site.

One potential use of AIEP would be to dissect the possible roles of GH3 enzymes in grape berry development. GH3-1 has been associated with the control of grape berry ripening, possibly by inactivating endogenous IAA through the formation of the non-cleavable IAA-Asp conjugate [33]. The expression of *GH3-6* in flowers and young berries [23] also indicates a role for this GH3 protein in berry development. Unlike *GH3-1* the expression of *GH3-6* in grape berries was repressed by auxin treatments in a previous study [23]. A similar response to auxin application has been reported for *GH3-9* from Arabidopsis, which has been linked to auxin redistribution in roots [26].

In order to determine the *in vivo* inhibitory properties of AIEP, IAA and auxin-conjugate levels were measured in young berries that had been exposed to the synthetic auxin NAA, the inhibitor and different concentrations of the inhibitor in combination with NAA. The test was performed on agar plates to facilitate the uptake of the compounds within a short period of time. A 2-fold decrease in IAA-Asp levels after 6 h and 24 h exposure to 20 µM of AIEP was suggestive of an inhibition of GH3 activities (Fig. 5B). It was not possible to judge the degree of inhibition since the detected IAA-Asp could either have been derived from *de novo* synthesis or it could have been residual conjugate that had been synthesised before the commencement of the experiment. Not much is known about the metabolic fate of IAA-Asp, but there is evidence that oxidation of the bound IAA can occur [17], [46] which leads to the irreversible inactivation of the auxin.

A significant inhibition of the formation of NAA-Asp that occurred in response to the incubation on 0.5 µM NAA medium could only be detected after 6 h at the highest inhibitor concentration (20 µM) tested (Fig. 5C). The reduced NAA-Asp levels demonstrated the *in vivo* inhibition of auxin-conjugate formation by AIEP and indicated that the constant uptake of NAA from the medium possibly resulted in high NAA concentration within the cells of the berry, which consequently reduced inhibition over time and at low inhibitor concentrations (Fig. 5C).

In conclusion, this study presents the synthesis and initial characterisation of the most potent inhibitor of the GH3 family of enzymes so far described. For the first time an inhibitor of GH3 activities has been shown to be effective *in vivo*. AIEP is expected to be a useful tool in the study of GH3 protein function, either as a complement to knockout and overexpression mutants in model species like Arabidopsis, or as a means to manipulate GH3 activities in non-model species such as grapevine where long generation times and/or transformation restrictions limit experimental manipulation.

## Materials and Methods

### Synthesis of AIEP

Solid NaHCO_3_ (593 mg, 7.06 mmol) was added to a stirring suspension of adenosine-5′-monophophate monohydrate (AMP.H_2_O; Sigma) (2.43 g, 6.65 mmol) in water (30 mL) and stirring was continued until complete dissolution of AMP was achieved. 3-(2-bromoethyl)indole (0.51 g, 2.23 mmol; Sigma) was added to the mixture as a solution in acetone (120 mL; Crown Scientific), which resulted in a white suspension. Water (ca. 5 mL) was added until the suspension dissolved completely. The resulting mixture was refluxed at ca. 60°C for 5 days, and then allowed to cool to room temperature. The aqueous mixture was extracted with CH_2_Cl_2_ (4×50 mL) and then freeze-dried to provide a yellow powder. The crude mixture was purified by repeated separation of approximately 200 mg batches using preparative HPLC using a binary pump system (Lab Alliance Series II pumps). Prior to separation, HP-20SS (50 mL) was equilibrated by sequential washings with 200 mL each of water, MeOH and acetonitrile, and then flushed with 200 mL of water. Each batch was separated using the following conditions: flow rate 9 mL/min; solvent A, acetonitrile, solvent B, water; 0–15 min, 5% A; 15→35 min, 5→30% A; 35→55 min, 30→100% A. Eluting compounds were monitored by UV detection (280 and 210 nm) (Thermo Model 525 UV-Vis detector). A large peak corresponding to AIEP at 24→31 min was collected. Between each batch, the HP-20SS was washed at 9 mL/min with water for 20 min. The combined 24→31 min fractions for each batch were then pooled and freeze-dried to provide 220 mg (20%) of a white, fluffy powder. NMR (Bruker Avance III 600 MHz): ^1^H NMR (600 MHz, d6-DMSO/D_2_O 1∶1) 8.47 (s, 1H, AMP aromatic), 8.13 (s, 1H, AMP aromatic), 7.41 (d, 1H, *J* = 7.9 Hz, indole aromatic), 7.29 (d, 1H, *J* = 8.1 Hz, indole aromatic), 7.08 (s, 1H, indole aromatic), 7.00 (app t, 1H, *J* = 7.5 Hz, indole aromatic), 6.86 (app t, 1H, *J* = 7.4 Hz, indole aromatic), 5.92 (d, 1H, *J* = 5.8 Hz, ribose O-C**H**-N), 4.55 (app t, 1H, *J* = 4.5 Hz, ribose CH_2_-C**H**O-CHOH), 4.17 (m, 1H, ribose C**H**-OH), 4.04 (m, 1H, ribose C**H**-OH), 3.87–3.82 (m, 4H, ribose C**H_2_** and indole-ethyl C**H_2_**), 2.87 (t, 2H, *J* = 7.5 Hz, indole-ethyl C**H_2_**). ^13^C NMR (150 MHz, d6-DMSO/D_2_O 1∶1) 156.2, 153.7, 150.1, 140.2, 136.5, 127.8, 123.4, 121.6, 119.1, 119.0 (×2), 111.9, 111.4, 87.3, 84.6, 74.5, 71.2, 65.3, 64.9, 27.2. LRMS (Waters 39 Synapt HDMS fitted with an Analytica electro spray source (ESI)), (ESI +ve mode): 491 (100%), 402 (5%), 348 (70%), 279 (10%), 218 (45%). HRMS (ESI +ve mode): Calculated for C_20_H_24_N_6_O_7_P ([M+H]^+^) = 491.1444, found = 491.1447. Mpt. 181–185°C (dec.).

### Plant Material

For the *ex planta* berry experiment, grape berries (*Vitis vinifera* L. cv Shiraz) were sampled from a vineyard in the Adelaide Hills, South Australia (35.018223, 138.838220) 5 weeks before the initiation of ripening (10 January 2011) between 0900–1000 hrs and kept on ice until used.

### Ex Planta Berry Assay

Shiraz berries were sampled, sterilized and cut as described in [23]. Berries (20/plate, 3 replicates) were placed on petri dishes filled with 25 mL of Gamborg's media, 0.025% (w/v) Casein hydrolysate, 0.8% (w/v) agar, pH 5.7–5.8 and one or more of the following additives (final concentrations): NAA (Gibco BRL Life Technologies) (0.5 µM), AIEP (5 µM, 10 µM, 20 µM), 3% (w/v) sucrose. After 6 h or 24 h in the dark on parafilm sealed plates the berries were harvested, deseeded and frozen in liquid nitrogen.

### Chemical Synthesis of Labelled Auxin Amino Acid Conjugates

[Indole-D_5_]IAA, [indole-D_5_]IAA-Asp and NAA-[1,4-^13^C_2_]Asp were synthesised as described previously [23].

### Protein Purification and GH3 Enzyme Assay

The coding region of *GH3-6* was amplified by PCR from a Cabernet Sauvignon berry cDNA template using gene-specific primers (5′-TAT**CATATG**TTGCTAAGCTGTGATCCACATGA-3′, 5′- ATA**GCGGCCGC**TTTTGTTTCCATTTTGAAAGGC-3′) with additional *NdeI* and *NotI* sites (in bold). Cloning, heterologous expression and purification of GH3-6-His (C-terminal fusion) were essentially performed as described by [33], but instead of using PD-10 columns for desalting protein fractions were desalted and concentrated using 50 kDa cutoff filter units (Millipore). The TLC-based assays for IAA-amino acid conjugate formation were performed as described by [33].

### Determination of Kinetic and Inhibition Parameters

The identification of suitable protein amounts and incubation times to be used for the determination of steady-state kinetic parameters for GH3-6 was done as previously described for GH3-1 and GH3-2 [23] with Asp as the conjugating amino acid. Initial velocity studies for GH3-1 and GH3-6 were determined using standard assay conditions [23] but with varying concentrations of either MgATP (0–1000 µM) or IAA (0–1000 µM). Reactions were stopped after 10 min ensuring the synthesis of the IAA-Asp product within the linear range of detection, followed by the addition of labelled standards, sample extraction and product quantitation using LC-ESI-MS/MS as described in [23]. All data were analysed using SigmaPlot 11.0 software. *K_m_*- and *V_max_*-values were determined by fitting data in Fig. 2 to the Michaelis-Menten equation. For inhibitor studies 0.1–50 µM AIEP were added to the reaction mixtures (Fig. 2) and the modes of inhibition as well as *K_i_*-values were established using Dixon plots (Fig. 4) [47].

### LC-ESI-MS/MS Analysis of Auxins and Conjugates

For the quantification of IAA, IAA-Asp and NAA-Asp in grapes, auxins were extracted from 100 mg of grape berry tissue spiked with 500 pmol of [Indole-D_5_]IAA, [Indole-D_5_]IAA-Asp and NAA-[1,4-^13^C_2_]Asp as internal standards. Extraction, purification and LC-ESI-MS/MS analysis were carried out as described previously [23] with the following changed solvent conditions: 0–16 min isocratic 60% (v/v) MeOH, linear gradient from 60% (v/v) to 95% (v/v) MeOH in 1 min, held for 6 min, from 95% (v/v) to 60% (v/v) in 1 min, held for 6 min, 0.4 mL min^−1^. The analysis of conjugates produced by the *in vitro* reactions with recombinant GH3-1 and GH3-6 was performed as described in [23] using the following solvent conditions for all assays: 0–8 min isocratic 60% (v/v) MeOH, linear gradient from 60% (v/v) to 95% (v/v) MeOH in 1 min, held for 5 min, from 95% (v/v) to 60% (v/v) in 1 min, held for 6 min, 0.4 mL min^−1^.

### Statistical Data Analysis

All statistical analyses were performed using SPSS 15.0 (SPSS, Chicago, Illinois, USA).
